# Molecular Characterization of Annexin B2, B3 and B12 in *Taenia multiceps*

**DOI:** 10.3390/genes9110559

**Published:** 2018-11-19

**Authors:** Cheng Guo, Yue Xie, Yuchen Liu, Ning Wang, Jiafei Zhan, Xuan Zhou, Christiana Angel, Xiaobin Gu, Weimin Lai, Xuerong Peng, Guangyou Yang

**Affiliations:** 1 Department of Parasitology, College of Veterinary Medicine, Sichuan Agricultural University, Chengdu 611130, China; Chengguo_92@hotmail.com (C.G.); xyue1985@gmail.com (Y.X.); liuyuchen1229@163.com (Y.L.); wangningzhuhui@hotmail.com (N.W.); zhanjiafei@hotmail.com (J.Z.); qazi5502@yahoo.com (C.A.); guxiaobin198225@126.com (X.G.); aliciaheml@hotmail.com (W.L.); 2 Institute of Animal Genetics and Breeding, College of Animal Science and Technology, Sichuan Agricultural University, Chengdu 611130, China; zhouxuan198866@163.com; 3 Department of Veterinary Parasitology, Faculty of Veterinary Sciences, Shaheed Benazir Bhutto University of Veterinary and Animal Sciences, Sindh 67210, Pakistan; 4 Department of Chemistry, College of Life and Basic Science, Sichuan Agricultural University, Chengdu 611130, China; pxuerong@aliyun.com

**Keywords:** *Taenia multiceps*, annexins, prokaryotic expression, immunofluorescence localization, qRT-PCR

## Abstract

*Coenurus cerebralis*, the metacestode of *Taenia multiceps*, causes coenurosis, a disease severely affecting goat, sheep, cattle and yak farming and resulting in huge economic losses annually. Annexins bind calcium ions and play an important role in flatworm parasite development. To explore potential functions of annexins in *T. multiceps*, three homologous genes, namely, *TmAnxB2*, *TmAnxB3* and *TmAnxB12*, were screened from the transcriptome dataset, amplified from *C. cerebralis* cDNA and subjected to bioinformatics analysis. Then, polyclonal antibodies recognizing the recombinant TmAnxB2 (rTmAnxB2) and rTmAnxB3 were prepared for localization of TmAnxB2 and TmAnxB3 in different tissues and developmental stages by immunofluorescence. The transcription of all three genes was also measured by relative fluorescent quantitative PCR. The sizes of rTmAnxB2, rTmAnxB3 and rTmAnxB12 were 58.00, 53.06 and 53.51 kDa, respectively, and rTmAnxB12 was unstable. Both rTmAnxB2 and rTmAnxB3 were recognized by goat-positive *T. multiceps* sera in Western blots. Immunofluorescence revealed that TmAnxB2 and TmAnxB3 were localized in the protoscolex and cyst wall and TmAnxB3 was also detected in adult cortex. TmAnxB2 and TmAnxB12 mRNA levels were determined to be highest in oncospheres and protoscolex, whereas transcription of TmAnxB3 was highest in scolex and immature segments. Taken together, these findings indicate that TmAnxB2 and TmAnxB12 may play critical roles in *T. multiceps* larvae, while TmAnxB3 may have important functions in adults. These results will lay the foundation for functional research of annexins in *T. multiceps*.

## 1. Introduction

*Taenia multiceps* is a flatworm parasite that is widely distributed around the world, and its eggs enter the environment via feces of its definitive hosts such as dogs, wolves, foxes and other canids [1,2,3]. Intermediate hosts, such as goats, sheep, cattle and yaks, are infected with coenurosis following ingestion of pasture containing parasite eggs. Metacestodes of *T. multiceps*, known as *Coenurus cerebralis*, then parasitize the central nervous system and intermuscular, subcutaneous, and liver tissues of these intermediate hosts [4,5,6] and cause clinical manifestations such as circling symptoms, depression and even death [7,8], which lead to serious economic losses to farming, especially in economically developing countries within Africa and Southeast Asia [9,10,11]. Furthermore, this parasite also causes zoonotic infections in humans and poses severe threats to public health [12,13,14].

Annexins are a class of phosphatide binding proteins with calcium binding activity. These proteins are widely expressed in all eukaryotes and play important roles in various biological activities such as cell membrane transport, signal transduction, and calcium channel formation [15,16]. Furthermore, these proteins have been known to regulate the biological responses related to inflammation and help to maintain the stability of biofilm structure [15,16,17]. In parasites, annexins are thought to have important functions in maintaining cell structural integrity and regulating parasites to host immune responses [18]. However, there is very little information available on annexins in the parasitic cestode *T. multiceps*. In the present study, based on available transcriptome data of adult *T. multiceps* that were sequenced in our laboratory [12], we screened three annexin homologous genes, namely, *TmAnxB2*, *TmAnxB3* and *TmAnxB12*, and further amplified and expressed these three recombinant proteins TmAnxB2 (rTmAnxB2), rTmAnxB3 and rTmAnxB12 using a prokaryotic expression system. Due to instability of the rTmAnxB12, only TmAnxB2 and TmAnxB3 proteins were investigated for their localization in different tissues and developmental stages of *T. multiceps* by immunofluorescence. Meanwhile, the transcriptional differences of all three genes were also analyzed by the relative fluorescent quantitative PCR. These findings will lay a foundation for the future functional analysis of annexins in *T. multiceps*.

## 2. Materials and Methods

### 2.1. Ethical Approval

This study was reviewed and approved by the Animal Ethics Committee of Sichuan Agricultural University (AECSCAU; Approval No. 2011-028). Animals were handled strictly in accordance with the animal protection law of the People’s Republic of China (released on 18 September 2009) and the National Standards for Laboratory Animals in China (executed on 1 May 2002).

### 2.2. Parasites and Animals

Coenuri larvae were isolated from the brains of naturally infected goats at a slaughterhouse in Sichuan Province, China. Adult worms were derived from a four-month-old beagle dog infected with *C. cerebralis* cysts at 45 days post-infection. The dog was kept alone in a clean cage, with clean food and water supplied ad libitum. Gravid proglottids from dog feces were collected before sacrifice. *T. multiceps* oncospheres were obtained as previously described [19]. All parasite materials were washed four times with sterile saline and stored in liquid nitrogen or 4% paraformaldehyde. In addition, *T. multiceps*-positive and negative goat serum samples were provided by the Department of Parasitology, College of Veterinary Medicine, Sichuan Agricultural University (Sichuan, China).

### 2.3. Bioinformatics Analysis

The open reading frames (ORFs) of genes encoding TmAnxB2, TmAnxB3 and TmAnxB12 were predicted using ORF finder with 300-bp minimal ORF in length and “ATG only” of ORF start codon (http://www.ncbi.nlm.nih.gov/gorf/gorf.html). Basic physicochemical properties and stability coefficients of these annexins were predicted by ProtParam with default parameters (http://web.expasy.org/protparam/), and signal peptides were assessed using SignalP (http://www.cbs.dtu.dk/Services/SignalP/). BaCelLo (http://gpcr.biocomp.unibo.it/bacello/pred.htm) was used to predict the subcellular localization of the three proteins, and the protein secondary was predicted using the NPSA_server (http://npsa-pbil.ibcp.fr/). Tertiary (3D) structures were modeled through SWISS-MODEL (http://swissmodel.expasy.org/) and by referring to the 2.5 Å resolution crystal structure of *Schistosoma mansoni* annexin (PDB accession No. 4MDV) for TmAnxB2, 1.42 Å crystal structure of annexin V (PDB accession No. 1yii.1.A) for TmAnxB3 and 2.8 Å crystal structure of annexin XII (PDB accession No. 1aei.1.A) for TmAnxB12. Phosphorylation sites were predicted by Motif Scan with default parameters (https://myhits.isb-sib.ch/cgi-bin/motif_scan), and phylogenetic analysis was conducted using maximum-likelihood (ML) method and plotted by PhyML 3.1 (http://www.atgc-montpellier.fr/phyml/versions.php) using the Jones–Taylor–Thornton (JTT) model selected under the AIC criterion by ProtTest 3 (http://darwin.uvigo.es/our-software/). Support for nodes was estimated by analyzing 1000 bootstrap replicates for each locus.

### 2.4. Cloning, Expression and Purification of Recombinant TmAnxB2, TmAnxB3 and TmAnxB12

Total RNA was extracted from oncospheres, coenuri, scolex, immature proglottids, mature proglottids, and gravid proglottids using an RNA Extraction Kit (Tiangen, Beijing, China) according to the manufacturer’s instructions, and template cDNAs were synthesized using a RevertAid First Strand cDNA Synthesis Kit (Fermentas, ON, Canada). Based on the assembled *T. multiceps* transcriptome dataset (Unigene 18519) and the AnxB2 sequence of *Taenia solium* (GenBank accession No. AY998562.1), specific primers for *TmAnxB2* were designed. Primers for *TmAnxB3* were designed based on the *T. multiceps* transcriptome dataset (Unigene 19512) and the AnxB3 sequence of *T. solium* (GenBank accession No. DQ010543.1). Gene-specific primers for *TmAnxB12* were designed using the *T. multiceps* transcriptome dataset (Unigene 17682) and an annexin from *Echinococcus granulosus* (GenDB No. EgrG_000237700). All three pairs of primers are listed in Table 1. Notably, apart from these three TmAnxBs, no other homologs were found within such *T. multiceps* transcriptome. After PCR amplifications, the products were digested with restriction enzymes, gel-purified and sub-cloned into the pET32a (+) expression vector, and the resultant constructs were transformed into competent *Escherichia coli* BL21 (DE3) cells. *E. coli* cells containing pET32a-TmAnxB2, pET32a-TmAnxB3 and pET32a-TmAnxB12, respectively, were cultivated at 37 °C for 4 h, induced with 1 mM isopropyl-β-d-1-thiogalactopyranoside (IPTG), and cultured for further 6 h. Then, the recombinant TmAnxB2 (rTmAnxB2), rTmAnxB3 or rTmAnxB12 with a ≈18-kDa His-tag protein was harvested and purified using Ni2+ affinity chromatography with NGC 10 system (Bio-Rad Laboratories, Inc., Hercules, USA). Protein purity was analyzed by 12% sodium dodecyl sulphate-polyacrylamide gel electrophoresis (SDS-PAGE).

### 2.5. Western Blotting

Given that the purified rTmAnxB12 was unstable, only rTmAnxB2 and rTmAnxB3 were included in the Western blotting and subsequent immunohistochemical localization. Along with total worm extract, rTmAnxB2 and rTmAnxB3 were separated by 12% SDS-PAGE and transferred onto a nitrocellulose (NC) filter membrane (Bio-Rad). Membranes were washed three times with TRIS-buffered saline Tween-20 buffer, blocked with 5% (*w*/*v*) skim milk solution in phosphate-buffered saline (PBS) for 2 h at room temperature, and *T. multiceps*-positive goat serum (1:100 *v*/*v* dilution in PBS buffer) was added and incubated overnight at 4 °C. After three washes, horseradish peroxidase (HRP)-conjugated rabbit anti-goat IgG (Bio-Rad) diluted in PBS (1:3000 *v*/*v* dilution) was added and incubated at room temperature for 2 h. After four washes, blots were visualized using an Enhanced HRP-DAB Chromogenic Substrate Kit (Tiangen) according to the instructions.

### 2.6. Fluorescence Immunohistochemistry

Fresh worm tissues including oncospheres, coenuri, scolex, immature proglottids, mature proglottids, and gravid proglottids of *T. multiceps* were embedded in paraffin and sectioned at 5 μm thickness. After deparaffinization, slices were placed in citrate buffer (0.01 M, pH 6.0) at 95 °C for 15 min. After three washes with PBS buffer (0.01 M), 3% H_2_O_2_ was added to the slices and incubated at 37 °C for 30 min. After three washes, rabbit anti-rTmAnxB2 or anti-TmAnxB3 IgG as well as naïve rabbit serum diluted with PBS (1:100 *v*/*v*) were added to slices and incubated at 4 °C for 14 h. Fluorescein isothiocyanate (FITC)-conjugated goat anti-rabbit IgG diluted 1:100 in 0.1% Evans Blue was then incubated with the slices at 37 °C in a dark environment for 1 h. After washing with PBS, stained samples were viewed under an Olympus BX50 fluorescence microscope (Olympus, Tokyo, Japan) using the red light as its sole light source.

### 2.7. Quantitative Real-Time PCR

Expression profiles of these three homologous *TmAnxB2*, *TmAnxB3* and *TmAnxB12* genes in oncospheres, protoscolex, scolex, immature proglottids, mature proglottids, and gravid proglottids were probed using quantitative real-time PCR (qRT-PCR). The actin gene was simultaneously used as an internal control for normalization. The qRT-PCR was performed using a CFX manager (Bio-Rad) according to the manufacturer’s instructions. The cycling conditions comprised a pre-incubation at 95 °C for 30 s, followed by 40 cycles at 95 °C for 5 s, 57.4 °C /55.7 °C for 20 s, and 72 °C for 20 s. The primer sequences are provided in Table 2. Relative expression values were calculated using the 2^−ΔΔCt^ method. Each gene was tested in quadruplicate.

## 3. Results

### 3.1. Molecular Features of Taenia multiceps Annexins

**Figure 1:**
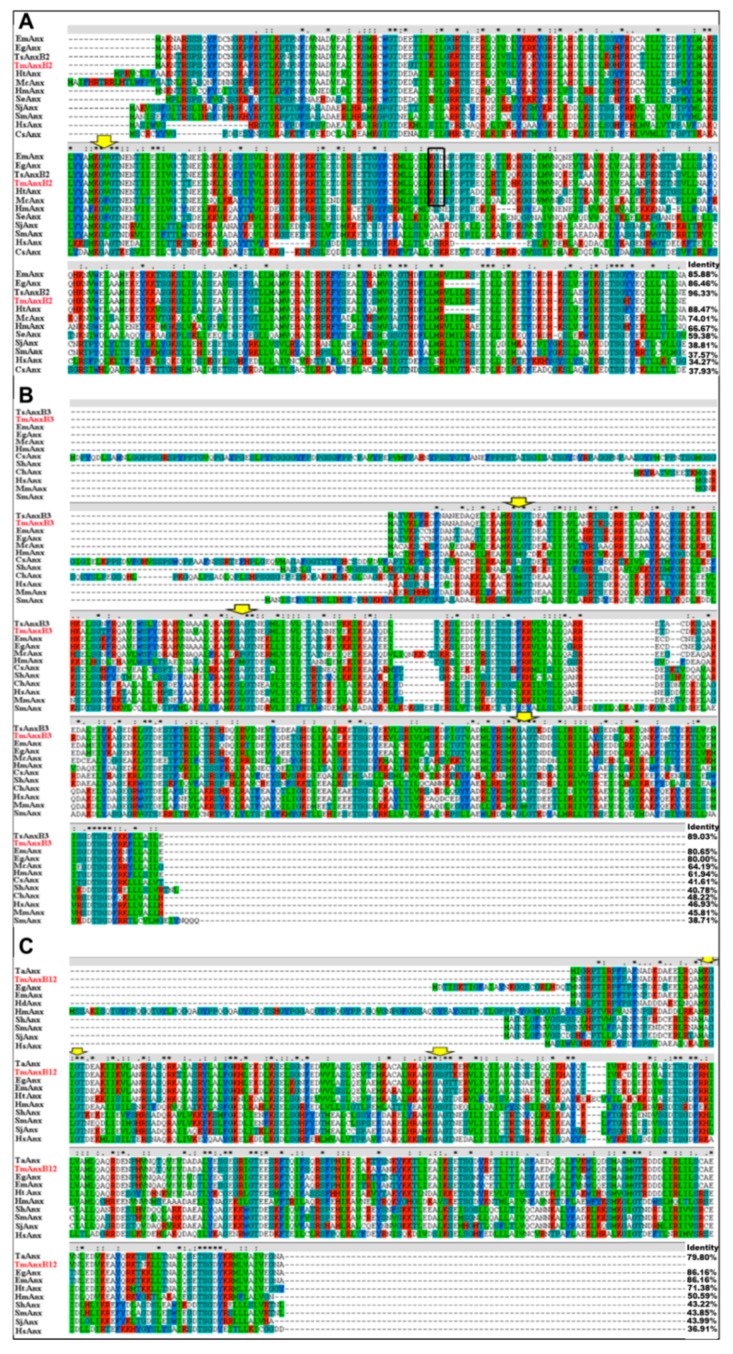
Sequence alignment analyses of: AnxB2 (**A**); AnxB3 (**B**); and AnxB12 (**C**) in *T. multiceps* and other species. (**A**) The following sequences were retrieved from the GenBank protein sequence database (accession numbers are indicated in parentheses): Em, *Echinococcus* *multilocularis* (CDI98110.1); Eg, *Echinococcus granulosus* (CDS21833.1); Ts, *Taenia solium* (AAY17503.1); Ht, *Hydatigena taeniaeformis* (A0A0R3X1T1); Mc, *Mesocestoides corti* (A0A0R3UMC3); Hm, *Hymenolepis microstoma*(CDS30725.1); Se, *Spirometra erinaceieuropaei* (ADM26238.1); Sj, *Schistosoma japonicum* (AAW25344.1); Sm, *Schistosoma mansoni* (AAC79802.3); Hs, *Homo sapiens* (AAH00871.1); Cs, *Clonorchis sinensis*(GAA48684.1); (**B**) The following sequences were retrieved from the GenBank protein sequence database (accession numbers are indicated in parentheses): Ts, *Taenia solium* (AAY27744.1); Em, *Echinococcus multilocularis* (CDI98572.1); Eg, *Echinococcus granulosus* (EUB59082.1); Mc, *Mesocestoides corti* (A0A0R3UJ25); Hm, *Hymenolepis microstoma* (CDS27549.1); Cs, *C. sinensis* (GAA33818.2); Sh, *Schistosoma haematobium* (KGB40261.1); Ch, *Capra hicus* (XP_013824596.1); Hs, *Homo sapiens* (CAG46637.1); Mm, *Mus musculus* (NP_081487.1); Sm, *S. mansoni* (AAC79802.3); (**C**) The following sequences were retrieved from the GenBank protein sequence database (accession numbers are indicated in parentheses): Ta, *Taenia asiatica* (A0A0R3W340); Eg, *E. granulosus* (EUB63408.1); Em, *Echinococcus multilocularis* (CDI98522.1); Hd, *Hymenolepis diminuta* (A0A0R3SPK4); Hm, *Hymenolepis microstoma*(CDS26802.2); Sh, *Schistosoma haematobium* (XP_012800019.1); Sm, *S. mansoni* (XP_018651719.1); Sj, *Schistosoma japonicum* (CAX69693.1); Hs, *Homo sapiens* (AAH00871.1). The yellow arrow in the figure represents the K-G-X-G-T sequence; the black box is the KGD sequence.

Sequencing analysis showed that genes for TmAnxB2, TmAnxB3 and TmAnxB12 contained ORFs of 1065 bp, 933 bp and 957 bp, respectively, encoding predicted polypeptides of 354, 310 and 318 amino acids, respectively. None of the annexins showed signal peptides or transmembrane domains, however, the signature sequence K-G-X-G-T was observed in all these three annexins. The predicted molecular weights of TmAnxB2, TmAnxB3 and TmAnxB12 were 40.00, 35.06 and 35.51 kDa, respectively, and the predicted isoelectric points (pIs) were 6.42, 5.53 and 6.14, respectively. In addition, the stability of these proteins was also predicted, and the results showed that a higher instability index (40.05) was observed in TmAnxB12, indicating the relative instability of this protein. Similarity comparison of three annexins revealed a sequence similarity of 27.89–42.77% at the amino acid level. Multiple sequence alignment revealed that TmAnxB2 shares 96.33% identity with AnxB2 from *T. solium* (TsAnxB2; GenBank accession No. AAY17503.1), 86.46% with *E. granulosus* annexin (GenBank accession No. CDS21833.1), and 85.88% with *Echinococcus multilocularis* annexin (GenBank accession No. CDI98110.1). TmAnxB3 shares 89.03% identity with AnxB3 from *T. solium* (TsAnxB3; GenBank accession No. AAY27744.1), 61.94–80.65% identity with other Taeniidae annexins, and only 38.71% identity with *S. mansoni* annexin (GenBank accession No. AAC79802.3). TmAnxB12 shares more than 85% identity with its orthologs in *E. granulosus* and *E. multilocularis*, and 43.22–43.99% identity with the equivalent proteins in Trematoda (Figure 1).

**Figure 2:**
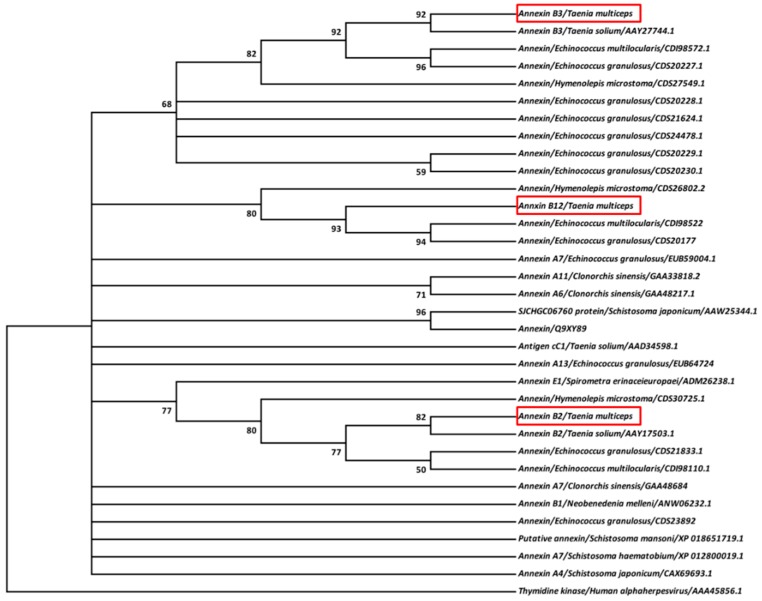
Phylogenetic analysis of annexin proteins. Phylogenetic analysis of the full-length amino acid sequences of *T. multiceps* annexins (in red boxes) and their homologs. The tree was constructed by the maximum-likelihood (ML) method and plotted with PhyML 3.1 with the Jones–Taylor–Thornton model selected by ProtTest 3 (http://darwin.uvigo.es/our-software/). Bootstrap values are indicated at the nodes (1000 replications). The scale indicates an estimate of substitutions per site, using the optimized model setting.

Based on sequence alignment of multiple annexins from Taeniidae and Trematoda, a ML phylogenetic tree was constructed, in which three annexins of *T. multiceps* were located on different branches. Specifically, TmAnxB2 and TmAnxB3 showed relatively close relationships to annexin B2 and B3 of *T. solium*, respectively; however, TmAnxB12 was more closely related to orthologs in *Echinococcus* spp. and *Hymenolepis* *microstoma* (Figure 2).

### 3.2. Structural Analysis of Taenia multiceps Annexins

**Figure 3:**
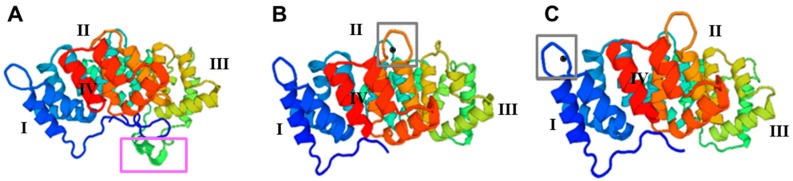
Predicted 3D structural models of: TmAnxB2 (**A**); TmAnxB3 (**B**); and TmAnxB12 (**C**). (**A**) The structure of TmAnxB2 is based on the crystal structure of *S. mansoni* Annexin (PDB accession code 4mdu1.A); (**B**) The structure of TmAnxB3 is based on the crystal structure of chicken annexin V (PDB accession code 1yii.1.A); (**C**) The structure of TmAnxB12 is based on the crystal structure of annexin XII (PDB accession code 1aei.1.A). The repeats in core domains of three molecules are coloured blue (repeat I), cyan (repeat II), yellow (repeat III) and red (repeat IV). The N-terminal domains are coloured sapphire blue, and the I/II linker regions are shown in sea green, the II/III linker in green and the III/IV linker in orange. Black spheres indicate the calcium ions.

Using the SWISS-MODEL database, TmAnxB2, TmAnxB3 and TmAnxB12 were modeled, and their predicted tertiary structures are depicted in Figure 3. Structural analysis revealed a common repeated alpha-helical domain. In addition, a unique α-helical element between repeats II and III was observed in the 3D structure of TmAnxB2 (purple box in Figure 3A), but not in TmAnxB3 or TmAnxB12. Further analysis revealed that both TmAnxB3 and TmAnxB12 have calcium binding sites in domains III and I, respectively, unlike TmAnxB2. Specifically, a type II calcium ion binding motif (K-G-X-G-T-D-E-38 amino acid residues-D/E) was found in the repeat domain III of TmAnxB3, and a type III calcium binding motif (K-G-X-G-T-D-E) was observed in the repeat domain I of TmAnxB12 (grey boxes in Figure 3B,C), while a KGD motif was found between repeat domains II and III of TmAnxB2 instead of a calcium ion binding region. These observations therefore highlight that the structures of TmAnxB3 and TmAnxB12 are more similar to each other than to TmAnxB2.

TmAnxB2, TmAnxB3 and TmAnxB12 display typical characteristics of members of the annexin family, and have four repetitive domains of 57–72 amino acids. TmAnxB2 also has one tyrosine kinase phosphorylation site, two cAMP- and cGMP-dependent protein kinase phosphorylation sites, three protein kinase C phosphorylation sites, and four casein kinase II phosphorylation sites. TmAnxB3 has one cAMP- and cGMP-dependent protein kinase phosphorylation site, one tyrosine kinase phosphorylation site, six protein kinase C phosphorylation sites, and twelve casein kinase II phosphorylation sites. TmAnxB12 has four protein kinase C phosphorylation sites, 10 casein kinase II phosphorylation sites, and one protease weight complex region. Furthermore, both TmAnxB3 and TmAnxB12 have an EF-hand calcium ion binding region (grey boxes in Figure 3B,C) that is absent in TmAnxB2.

### 3.3. Expression, Purification and Western Blotting of Taenia multiceps Annexins

**Figure 4:**
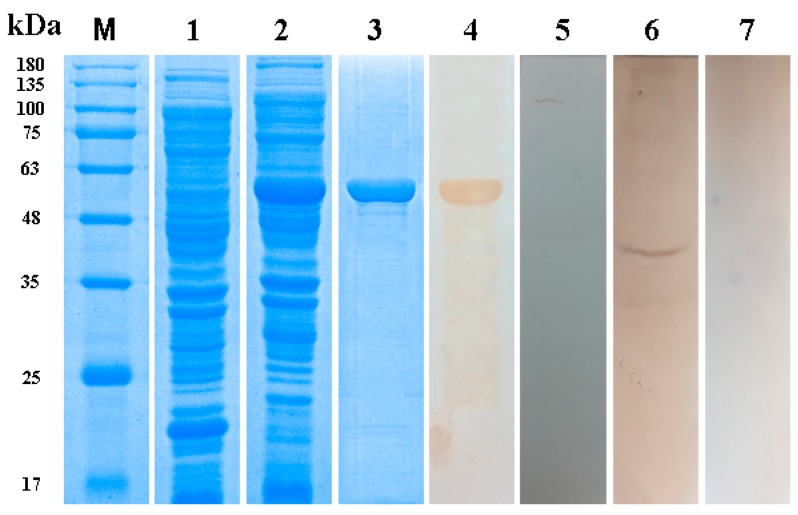
Purification and Western blotting of recombinant rTmAnxB2. M, Protein markers; 1, pET32a-vector; 2, pET32a-TmAnxB2; 3, Purified rTmAnxB2; 4, rTmAnxB2 detected by *T. multiceps*-positive goat serum; 5, rTmAnxB2 detected by *T. multiceps*-negative goat serum; 6, Western blot of *T. multiceps* extracts detected by rabbit anti-rTmAnxB2-IgG; 7, Western blot of *T. multiceps* extracts detected by healthy rabbit serum (controls).

**Figure 5:**
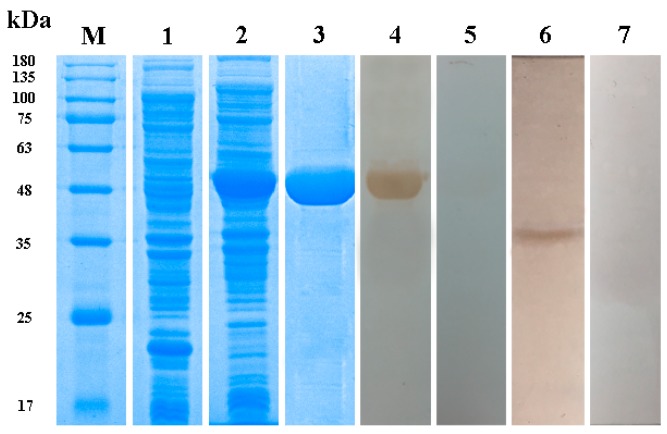
Purification and Western blotting of recombinant rTmAnxB3. M, Protein markers; 1, pET32a-vector; 2, pET32a-TmAnxB3; 3, Purified rTmAnxB3; 4, rTmAnxB3 detected by *T. multiceps*-positive goat serum; 5, rTmAnxB3 detected by *T. multiceps*-negative goat serum; 6: Western blot of *T. multiceps* extracts detected by rabbit anti-rTmAnxB3-IgG; 7, Western blot of *T. multiceps* extracts detected by healthy rabbit serum.

Recombinant rTmAnxB2, rTmAnxB3 and rTmAnxB12 were expressed with a ≈18 kDa His-tag, but rTmAnxB12 was unstable and readily degraded, consistent with the ProtParam prediction. Therefore, subsequent immunolocalization and Western blotting experiments were carried out only on rTmAnxB2 and rTmAnxB3. The molecular masses of rTmAnxB2 and rTmAnxB3 were ≈58 kDa and 53 kDa, respectively, close to the expected values. Rabbit anti-rTmAnxB2 and anti-rTmAnxB3 antibodies were prepared using purified rTmAnxB2 and rTmAnxB3, respectively. The native TmAnxB2 protein in adults was recognized using the rabbit anti-rTmAnxB2 antibody, yielding a single band of ≈40 kDa that was absent in the negative control (Figure 4). Likewise, the native TmAnxB3 protein in adults was recognized by the rabbit anti-rTmAnxB3 antibody, yielding a single band of ≈35 kDa (Figure 5). Furthermore, immunoblotting showed that both rTmAnxB2 and rTmAnxB3 were identified by *T. multiceps*-positive goat sera, suggesting the strong immunogenicity and immunoreactivity for both rTmAnxB2 and rTmAnxB3.

### 3.4. Immunolocalization of TmAnxB2 and TmAnxB3

**Figure 6:**
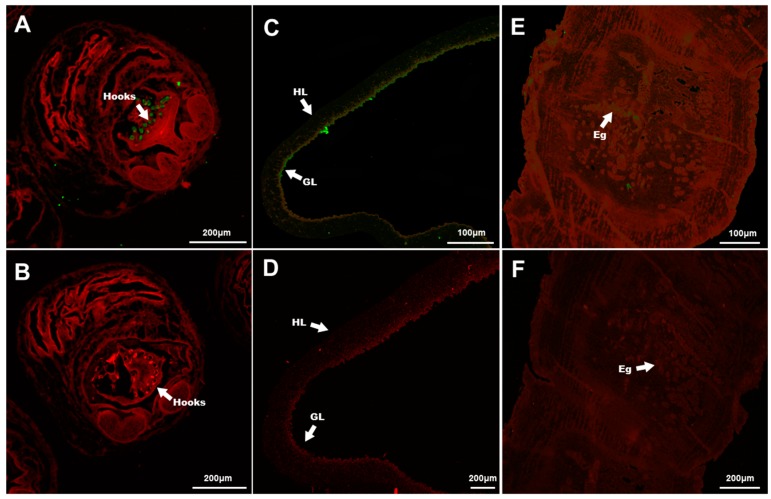
Immunofluorescence localization of TmAnxB2 in *T. multiceps*. Against the red background, the green fluorescent color shows the location of the native TmAnxB2 protein: (**A**) protoscolex with positive serum; (**B**) protoscolex with negative serum; (**C**) cyst wall with positive serum; (**D**) cyst wall with negative serum; (**E**) gravid proglottids with positive serum; and (**F**) gravid proglottids with negative serum; GL, germinal layer; HL, horny layer; Eg, eggs.

**Figure 7:**
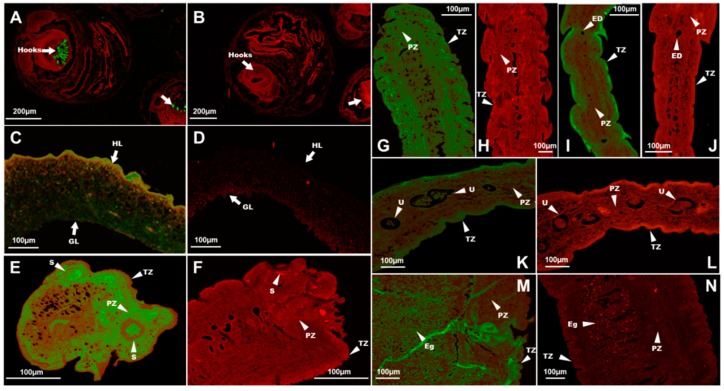
Immunofluorescence localization of TmAnxB3 in *T. multiceps*. Against the red background, the green fluorescent color shows the location of the native TmAnxB3 protein: (**A**) protoscolex with positive serum; (**B**) protoscolex with negative serum; (**C**) cyst wall with positive serum; (**D**) cyst wall with negative serum; GL, germinal layer; HL, horny layer; (**E**) scolex with positive serum; (**F**) scolex with negative serum; (**G**) neck with positive serum; (**H**) neck with negative serum; (**I**) immature proglottids with positive serum; (**J**) immature proglottids with negative serum; (**K**) mature proglottids with positive serum; (**L**) mature proglottids with negative serum; (**M**) gravid proglottids with positive serum; and (**N**) gravid proglottids with negative serum. Abbreviations: S, suckers; GL, germinal layer; HL, horny layer; TZ, tegument zone; PZ, parenchymatous zone; ED, excretory duct; U, Uterus; Eg, eggs.

Immunohistochemical analysis showed that TmAnxB2 and TmAnxB3 were distributed in the hooks of protoscolex and the germinal layers of the cyst wall of protoscolex (Figure 6 and Figure 7). However, compared to TmAnxB2, a larger amount of TmAnxB3 was present in the cyst wall of protoscolex. Notably, no fluorescence signal was detected for TmAnxB2 in the scolex, neck, immature proglottids, or mature proglottids of adults; however, a weak fluorescence signal was detected in the eggs of gravid proglottids (Figure 6). Conversely, a strong fluorescence signal was observed for TmAnxB3 in all segments of adults, indicating the evidence of tissue specificity. For instance, TmAnxB3 was highly localized in the tegument zone (TZ) of adults, widely distributed in the parenchymatous zone (PZ) and sucker of scolex, and present in all areas of the neck (Figure 7).

### 3.5. Transcriptional Profiles of Taenia multiceps Annexins

**Figure 8:**
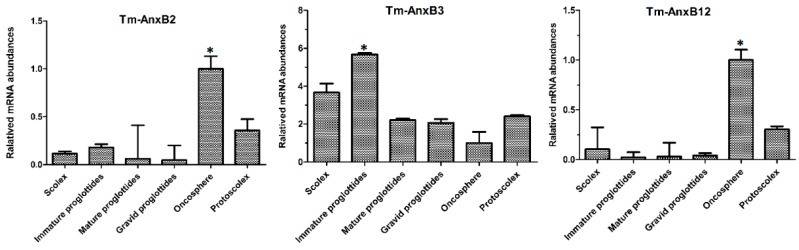
Differential transcription levels of TmAnxB2, TmAnxB3 and TmAnxB12 in different proglottids and developmental stages of *T. multiceps*. Levels of expression were determined using the ^ΔΔ^Ct method. Data are represented as the means ± SD (*n* = 3). Asterisks indicate fold changes that were statistically significant at *p* < 0.05.

The qRT-PCR results showed that TmAnxB2 was transcribed in both adults and larvae. As for the expression of TmAnxB2 in adults, no significant differences (*p* > 0.05) were observed in transcriptional level between the segments. However, the relative amount of TmAnxB2 mRNA in immature proglottids was 1.6-, 3- and 3.6-fold higher compared to scolex, mature proglottids and gravid proglottids, respectively. Meanwhile, in the larval stage, the mRNA levels of TmAnxB2 were highest in oncosphere, 2.78-fold higher than in protoscolex and 5–20 times higher than in the adults (*p* < 0.05; Figure 8). These results suggest that TmAnxB2 may play important roles in parasite development and host invasion of *T. multiceps* oncospheres.

Additionally, the TmAnxB3 gene was transcribed across all developmental stages of *T. multiceps*, with the highest expression in immature proglottids, 1.4–5.1-fold higher compared to other developmental stages or segments (*p* < 0.05). Interestingly, relative TmAnxB3 mRNA levels increased gradually from oncospheres to adults, suggesting that TmAnxB3 may be critical for *T. multiceps* development from larvae to adult stages (Figure 8). Similarly, TmAnxB12 gene expression was highest in oncospheres of *T. multiceps*, 3–50 times higher than in other stages and tissues (*p* < 0.05). Furthermore, relative TmAnxB12 mRNA levels in protoscolex were 3–15 times higher than in proglottids (*p* < 0.05, Figure 8). These findings indicate that TmAnxB12 may have an important role in growth and development of *T. multiceps*, and possibly the host invasion.

## 4. Discussion

Annexins are a multigene protein family with typical conserved sequence characteristics. Members of this family have four homologous repeat domains, each of ≈70 amino acid residues, and these domains generally include a typical K-G-X-G-T motif and a typical annexin type II binding site [20]. Structural prediction of TmAnxB2, TmAnxB3 and TmAnxB12 revealed that all these share the typical characteristics of annexin family members. However, TmAnxB2 appears to lack the calcium binding sites, which are replaced by a unique KGD motif in some repeats. This feature has been reported in other cestode homologs such as Ts-AnxB2, Eg-Anx, Em-Anx, Ht-Anx and Mc-Anx [21,22,23]. Nevertheless, it is worth noting that such structure was just modeled by referring to the available crystal structure of *S. mansoni* annexin and needs to be examined further in future. Moreover, the ML-based phylogeny placed these three annexin homologs of *T. multiceps* in different groups, in which TmAnxB2 and TmAnxB3 clustered with annexin B2 and B3 of *T. solium*, respectively. Interestingly, in a recent genome-based study, Cantacessi and colleagues showed that among six distinct annexin clades (I–VI) found across parasitic plathyhelminthes and nematodes, *T. solium* annexin B2 and B3 were positioned in Clade I [20]. Therefore, it is reasonable to infer that TmAnxB2 and TmAnxB3 of *T. multiceps* might also be positioned under such clade. Previous reports have demonstrated that protein phosphorylation plays a key role in parasitic flatworms, including coordination of differentiation of cells and organs and mediation of signal transduction [24,25,26,27]. Our predictions revealed numerous phosphorylation sites in TmAnxB2, TmAnxB3 and TmAnxB12, suggesting their potential implication in development and signal transduction. Moreover, by comparing the functional sites, we observed that TmAnxB3 has more protein kinase C phosphorylation sites and casein kinase II phosphorylation sites compared to TmAnxB2 or TmAnxB12. Meanwhile, TmAnxB3 was most highly expressed in adult *T. multiceps.* Evidence from previous studies indicates that protein kinase C and casein kinase II phosphorylation sites can play an important role in growth and development via catalytic phosphorylation of serine and threonine [24]. Similarly, our results suggest that TmAnxB3 may be an important protein having potential implication in growth and development in *T. multiceps*.

Annexins are calcium-dependent phospholipid binding proteins with calcium binding capacity that participate in various biological activities related to biofilm development via membrane phospholipid binding [15]. In parasites, *Leishmania* promastigotes bind annexin V to ensure the viability of promastigotes due to the lack of phosphatidylserine [28]. Meanwhile, annexin B1 of *T. solium* can induce calcium-dependent eosinophil apoptosis and diminish host immune responses [29,30,31]. Moreover, ANXB33 in *E. granulosus* is located in host inflammatory cells and fibroblasts, suggesting its potential involvement in reactions between hosts and parasites [21]. In the congeneric *T. solium*, previous studies showed that annexin B2 has a procoagulant effect in vitro, whereas annexin B1 binds to human eosinophils and generates a calcium current, thereby resisting attack by the host immune system [32,33]. In our present study, fluorescence immunolocalization showed that TmAnxB3 was expressed in both larvae and adults in *T. multiceps*, whereas TmAnxB2 was only expressed during the coenurus and oncosphere stages, suggesting that TmAnxB2 may be an important protein in the development of larvae, while TmAnxB3 may play a key role in the development of adult stages.

TmAnxB3 was mainly distributed in the tegument of each segment in *T. multiceps*, and was also widely distributed in the parenchymatous zone of the scolex and neck. These findings are consistent with the known distribution of annexins in *E. granulosus*, *Clonorchis sinensis* and *S. mansoni* [21,22,34]. The tegument of parasitic cestodes not only plays a key role in escaping the attack from host immune system, it is also an important organ for nutrient acquisition and signal transduction during long-term parasitic life [12]. In this study, TmAnxB3 was mainly distributed in the adult tegument, indicating its implication in nutrition and interaction between parasites and hosts. Furthermore, TmAnxB2 was mainly distributed in the cyst wall of *T. multiceps*, similar to AnxB1 in *T. solium* [30,35].

During parasitism, annexins can protect parasite tissues and help them escape attack from the host immune system [18]. Importantly, these proteins contribute to physiological functions in taeniidae in a tissue-specific manner [36,37]. For instance, AnxB30 of *C. sinensis* is transcribed at high levels in metacercaria and at lower levels in adults and eggs compared to other stages [34]. However, ANXB33 of *E. granulosus* is mainly transcribed in the germinal layer and protoscoleces, but not expressed in the cystic fluid or cyst wall [21]. In the present study, all three Tm annexins were expressed at relatively high levels in the metacestode stage, including oncosphere and coenurus stages. Notably, from coenurus to scolex, the relative transcription levels of TmAnxB2 and TmAnxB12 showed a downward trend, while the relative transcription of TmAnxB3 showed an upward trend. These findings suggest that TmAnxB3 may play a significant role in growth and development of adults. By contrast, TmAnxB2 and TmAnxB12 were transcribed at higher levels during the oncosphere period, and levels were statistically different from those in coenurus and scolex (*p* < 0.05), suggesting that these two proteins may play important roles in larvae of *T. multiceps*. Nevertheless, further studies are awaited to confirm these findings, but the recent report by Li et al. on genome of *T. multiceps* provides a comprehensive profile of annexins in this worm [1]. These enticing findings, to a certain extent, suggest that more annexin genes should be considered for elucidating their common and/or specific roles in different tissues (e.g., scolex-neck proglottids, immature-mature proglottids and gravid proglottids) and developmental stages (e.g., oncospheres, protoscolexes and adults) of *T. multiceps*. In addition, the development of tapeworms is dominated by the development of segments, hence newly emerged segments are closer to the scolex [38,39]. Similar to other metazoans, protein phosphorylation in tapeworms coordinates the differentiation of cells and organs [24], and the numerous phosphorylation sites in TmAnxB2, TmAnxB3 and TmAnxB12 observed in our study are consistent with this, suggesting the potential implication of annexins in regulating the important biological functions i.e., growth and development in *T. multiceps*.

## 5. Conclusions

In this study, the full-length cDNAs encoding three homologous annexins (TmAnxB2, TmAnxB3 and TmAnxB12) were identified and characterized in *T. multiceps*. From these, TmAnxB2 and TmAnxB3 proteins were localized in the oncosphere, protoscolex, scolex, immature proglottids, mature proglottids, and gravid proglottids, as demonstrated by fluorescence immunohistochemistry. Furthermore, TmAnxB2 was demonstrated to possibly play an important role in the larval stage of *T. multiceps*, while TmAnxB3 appears to be more important in adults. Taken together, these enticing findings of our study provide the reasonable basis for future studies focusing on exploration of potential functions of annexins in *T. multiceps*.

## Figures and Tables

**Table 1:** Primers used for PCR amplification of *Taenia multiceps annexins*.

Genes	Primers	Restriction Enzyme Sites	Reference Sequences
*TmAnxB2*	F: **CGC*GGATCC***ATGGCAAAAAATACTCGCTC	*Bam*HI	Unigene18519
	R: **CCG*CTCGAG***TTAGGATTCATTCAGTAGTGCG	*Xho*I	Ts AY998562.1
*TmAnxB3*	F: **CCG*GAATTC***ATGGCGACTGTCAAGCTTT	*Eco*RI	Unigene19512
	R: **CCG*CTCGAG***TCACTCCAGTATGGTGAGCA	*Xho*I	Ts DQ010543.1
*TmAnxB12*	F: **CGC*GGATCC***ATGAATGGGCGTCCAACTA	*Bam*HI	Unigene17682
	R: **CCG*GAATTC***TTATGCATTCCCCTCTACAA	*Eco*RI	EgrG_000237700

Note: Black and bold italics signify restriction enzyme sites.

**Table 2:** Primers used for quantitative real-time PCR (qRT-PCR) amplification of *T. multiceps* annexins.

Gene	Primers	Size (bp)
***TmAnxB2***	F: GGTTCAACACGCCGTAGACAGAC	101
	R: TGAGGACTCGCATGAGGAGGAAG	
***TmAnxB3***	F: TGCACCGCCACCAACAACG	114
	R: CACTCGCTTGAAGTCGCCAGAG	
***TmAnxB12***	F: AGGAGGTGACGGAGATGAAGGC	94
	R: GCAACGGCGATGATCTGGATGAG	
***Actin***	F: CTAAGGCGAACCGTGAGAAGATGAC	188
	R: GGCATGAGGCAAGGCGTAACC	
